# The protective effect and mechanism of dexmedetomidine in inhibiting ferroptosis

**DOI:** 10.3389/fphar.2025.1605363

**Published:** 2025-08-29

**Authors:** Xinyi Ren, Ran Wang

**Affiliations:** ^1^ Department of Anesthesiology, Kunshan Hospital of Chinese Medicine, Kunshan, Jiangsu, China; ^2^ Department of Anesthesiology, Second Affiliated Hospital of Soochow University, Suzhou, Jiangsu, China

**Keywords:** dexmedetomidine, ferroptosis, α_2_-AR agonist, oxidative stress, iron overload

## Abstract

Dexmedetomidine (DEX) is a highly selective α_2_-Adrenergic Receptor (α_2_-AR) agonist which inhibits sympathetic nerve activity, and has been shown to have a wide range of sedative, analgesic, anesthetic and other effects, as well as reducing inflammation and exerting neuroprotective functions. Researches show that DEX provides an advantage of protecting vital organs from injury, such as myocardial, kidney or cerebral injury. Nowadays, the regulatory effect of DEX in ferroptosis has become a headline in current researches. Ferroptosis is a type of programmed cell death discovered in recent years and is considered to play an important role in mediating the onset and progression of diseases. The aim of this review is to further clarify the role and mechanism of DEX in inhibiting ferroptosis.

## 1 Introduction

As a highly specific α_2_-AR agonist, DEX has approximately eight times higher affinity for α_2_-AR than that of clonidine (Weerink et al., 2017). DEX acts on the locus coeruleus, producing a concentration-dependent sedative effect, which allows patients to be aroused easily at low doses (0.2–0.3 ng/mL). With minimal impact on respiration and hemodynamics, making it widely used in clinical practice (Liu X. et al., 2021; Hu et al., 2022).

Studies have shown that even in neonate, DEX exhibits high tolerability and safety, with minor hemodynamic impact (Portelli et al., 2024; Curtis et al., 2023). Prolonged use of DEX for sedation in Intensive Care Units (ICU) can reduce the duration of mechanical ventilation, shorten hospital stays, and decrease the incidence of delirium (Chen et al., 2015). DEX can inhibit the release of substance P (a peptide mainly secreted by neurons) from the dorsal horn of the spinal cord, effectively alleviates acute pain, as well as reduces adverse reactions such as perioperative nausea and vomiting, agitation, and delirium (Cho et al., 2018; Grape et al., 2019; Bosch et al., 2023). DEX can reduce the expression of inflammatory factors and exert systemic anti-inflammatory effects (Mei et al., 2021). In rodent models, DEX reduces neuroinflammation by inhibiting the activation of microglial cells and the expression of pro-inflammatory cytokines (Yeh et al., 2018). Additionally, DEX has the capacity to reverse neuronal apoptosis and autophagy, thereby playing a neuroprotective role in the cerebral Ischemia/Reperfusion (I/R) model by antagonizing the Toll-Like Receptor 4 (TLR4) signaling pathway (Bozorgi et al., 2021).

The concept of ferroptosis, first proposed by Dixon in 2012, represents a mode of programmed cell death that is distinct from apoptosis (Dixon et al., 2012). The primary mechanism of ferroptosis involves the action of ferrous ion (Fe^2+^) or lipoxygenase, which catalyzes the unsaturated fatty acids in the cell membrane, leading to lipid peroxidation and, consequently, cell death (Fang et al., 2023). The toxicity of Fe^2+^ and lipid peroxidation were described as early as the 1950s (Bieri, 1959). As a highly conserved program, ferroptosis plays a crucial role in the physical development and diseases, significantly impacting multi-system diseases, including neurological, cardiac, hepatic, renal, gastrointestinal, pulmonary and pancreatic diseases (Tang D. et al., 2021). An increasing number of studies have focused on the potential pathogenic role and regulatory pathways of ferroptosis, a form of programmed cell death. Several reports have highlighted the inhibition effect of DEX on ferroptosis (Tao et al., 2022; Wang et al., 2020; Wang Z. et al., 2022). Therefore, the aim of this review is to further clarify the inhibitory effects of DEX on ferroptosis pathways and its protective effects on various organs. The relevant evidence mentioned above and the proposed ferroptosis mechanism or pathway involved are as follows (Table 1).

**TABLE 1:** Relevant ferroptosis mechanism or pathway.

Evidence	Mechanism	Reference
DEX reduces systemic inflammation and neuroinflammation in septic mice.	• Excessive lipid peroxidation is a significant trigger for ferroptosis, while abnormal inflammatory responses can lead to iron metabolism disorders and an imbalance in redox system. • Activation of the MAPK pathway promotes the production of pro-inflammatory cytokines IL-1β and IL-6, reduces the expression of GPX4, and triggers the occurrence of neuro inflammation and ferroptosis.	Mei et al. (2021)
DEX reduces lipopolysaccharide Induced neuroinflammation by inhibiting the production of pro-inflammatory cytokines IL-1β, TNF-α, and IL-6.		Yeh et al. (2018)
PI3K/Akt signaling pathways participate in the protection conferred by DEX against cerebral ischemia/reperfusion injury.	• PI3K/Akt signaling pathway enhanced GPX4 expression, while GPX4 is an important inhibitory protein of peroxidation, which can regulate the sensitivity of cells to ferroptosis.	Bozorgi et al. (2021)
Dietary selenium and cystine significantly reduce peroxidation in certain tissues of vitamin E-deficient chicks.	• Cystine/glutamate transporter and Glutathione peroxidase are involved in correcting redox imbalance in cells. • Glutathione is the major antioxidant in mammalian cells, and cysteine is the rate-limiting substrate for Glutathione biosynthesis.	Bieri (1959)
Excessive or defective ferroptosis can contribute to pathological cell loss, as well as to malignant processes.	• Tumor cells tend to be more iron-dependent when they are rapidly proliferating. In this case, tumor cells more sensitive to ferroptosis due to higher levels of intracellular iron and ROS. • NRF2 has a dual role in tumor progression: lack of NRF2 activity can lead to early tumorigenesis, whereas high basal NRF2 activity can trigger tumor progression and resistance to therapy.	Tang et al. (2021a)
DEX attenuates ferroptosis-mediated renal I/R injury and inflammation by inhibiting ACSL4 via α_2_-AR.	• ACSL4 remodels the phospholipid composition of cell membranes and promotes the interconnection between fatty acid metabolism and ferroptosis, determining cellular sensitivity to ferroptosis.	Tao et al. (2022)
DEX attenuates iron concentration and HO-1 overexpression, and enhances GPX4 expression.	• Overexpression of HO-1 can lead to excessive iron, which is detrimental to redox balance. • excess free iron can catalyze the formation of free radicals through the Fenton reaction, leading to oxidative stress and cellular damage. • GPX4 is an important inhibitory protein of peroxidation, which can regulate the sensitivity of cells to ferroptosis.	Wang et al. (2020)
DEX significantly alleviated myocardial infarction and decreased accumulation of Fe^2+^ and lipid peroxidation in cardiomyocytes. DEX significantly increased the expression levels of NRF2 and GPX4.	• Intracellular iron accumulation leads to the release of Fe^2+^ into the cytoplasmic labile iron pool and the occur of Fenton reaction, generating hydroperoxide radicals. • Unrestricted lipid peroxidation is a hallmark of ferroptosis. The unsaturation degree of the lipid bilayer is critical in determining cellular susceptibility to ferroptosis. • NRF2 is activated and translocates to the nucleus under oxidative stress conditions, increasing ferritin levels and activating Glutathione peroxidase.	Wang et al. (2022a)

Abbreviations: DEX, dexmedetomidine; MAPK, Mitogen-Activated Protein Kinase; IL-1β, Interleukin-1β; TNF-α, Tumor Necrosis Factor α; IL-6, Interleukin-6; GPX4, Glutathione Peroxidase 4; PI3K, Phosphatidylinositol 3-Kinase; Akt, Protein Kinase B; ROS, reactive oxygen species; NRF2, Nuclear factor erythroid 2-related factor 2; ACSL4, Acyl-CoA, synthetase long-chain family member 4; α_2_-AR, α_2_-Adrenergic Receptor; HO-1, Heme Oxygenase 1.

## 2 Characteristic of DEX

### 2.1 Route of administration and absorption

DEX was initially registered solely for intravenous administration. While intravenous administration of DEX acted rapidly, it also elevated the risks of bradycardia and sedation (Hung et al., 2023). In addition to this commonly used route of administration, previous studies have reported various alternative administration routes: DEX absorbs through the nasal and buccal mucosa, making it particularly suitable for uncooperative children. However, the onset time of intranasally is significantly slower than that of intravenously, and the duration of sedation is shorter, with a bioavailability estimated to be 40.7% (Pansini et al., 2021; Li et al., 2018; Lyu et al., 2022). DEX can be combined with local anesthetics as an adjunct to nerve blocks, which produces differential effects on sensory or motor nerves: prolonging the block time of sensory nerves but not motor nerves (Desai et al., 2021; Knych et al., 2022). The optimal dose of DEX during adjuvant nerve block is 50–60 μg, which greatly prolongs the duration of sensory block with the least adverse hemodynamic effects (Desai et al., 2021; Feng and Chen, 2023). Despite the low oral absorption of DEX, oral DEX (2.5–4 μg/kg) results in clinically satisfactory sedation, maintaining hemodynamic stability (Jen et al., 2024). In addition, DEX can also be administered via intrathecal, intramuscular and intra-articular routes (Weerink et al., 2017).

### 2.2 Distribution and elimination

The protein binding affinity of DEX is relatively high, with 94% of that bound to albumin or α1-glycoprotein in plasma (Morse et al., 2020). DEX distributes rapidly and extensively, easily crossing both the blood-brain and placental barriers. Normally, its distribution half-life is around 6 min. The apparent volume of distribution of DEX is body weight-dependent. In adults, the apparent volume of distribution ranges from 1.31 L/kg to 2.46 L/kg, the elimination half-life is 2.1–3.1 h, while the clearance rate is 0.6–0.7 L/min (Weerink et al., 2017; Portelli et al., 2024). DEX is primarily metabolized and cleared via glucuronidation and the cytochrome P450 system. The clearance mainly depends on hepatic blood flow, therefore, patients with hepatic function impairment need to use it with caution. The clearance rate of DEX in obese patients standardized by body weight is significantly lower than that in normal adults (Xu et al., 2017). DEX is mainly excreted through the kidney, but renal impairment has little impact on the pharmacokinetics of DEX (Gao and Wu, 2024).

### 2.3 Safety research

Previous studies have reported that DEX can be safely applied to children, even infants, producing effective sedation and analgesia without causing serious adverse events or withdrawal reactions and having a favorable safety profile (Portelli et al., 2024). Barends et al. (Barends et al., 2017) reported that the respiratory and hemodynamic safety of DEX was similar to that of midazolam. But patients treated with DEX has higher satisfaction rates and lower demand for analgesics, compared with midazolam. Goswami et al. (2021), yet reported that DEX provided a better safety profile as a preoperative drug compared to midazolam and was associated with a lower chance of delirium. DEX, unlike other sedatives or anesthetics, causes little respiratory depression, even using large doses. The impact of DEX on hemodynamics has long been controversial. On the one hand, DEX has the property of improving intraoperative hemodynamic stability and cardiovascular parameters (Motaghi et al., 2021). On the other hand, high-dose DEX infusion may lead to hemodynamic changes such as hypertension, hypotension, or bradycardia. This adverse effect is closely related to the loading dose and infusion rate, and its occurrence can be prevented by regulating the infusion dose and rate (Lee, 2019).

### 2.4 Organ protection

**FIGURE 1:**
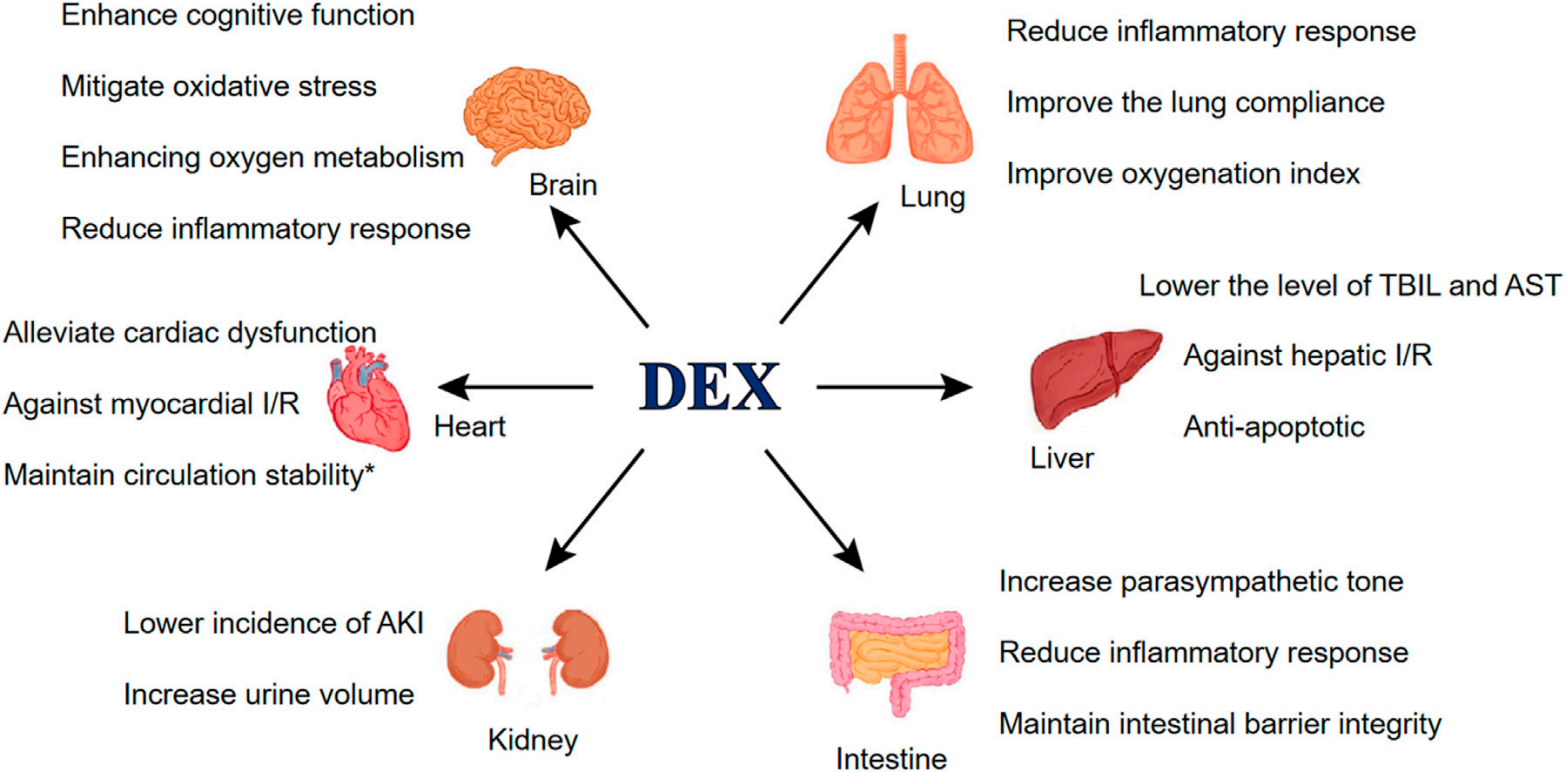
DEX’s protective effects. *postoperative complications mainly include cardiac arrest, atrial fibrillation, myocardial injury/infarction and heart failure. Abbreviations: I/R, Ischemia/Reperfusion; AKI, Acute Kidney Injury; TBIL, Total bilirubin; AST, Aspartate aminotransferase.

Since it came into use over two decades ago, numerous studies have elucidated the effects of DEX. As a commonly used anesthetic adjuvant in surgeries, DEX provides organ protection in various vital organ surgeries. Soh et al. (2020) reported that administration of DEX after anesthesia induction reduced the incidence of Acute Kidney Injury (AKI) after aortic surgery under cardiopulmonary bypass, which associates with a shorter length of hospital stay and a lack of adverse events. The renal protective effects of DEX have been validated in multiple studies (Liu et al., 2024; Zhu et al., 2020; Sun et al., 2021). In addition, DEX reduced Blood Urea Nitrogen (BUN) levels within 48 h postoperatively and significantly increased intraoperative urine output in patients (Qian et al., 2025). DEX is also commonly used in patients undergoing cardiac surgery due to its protective effects on myocardial against I/R injury and its ability to reduce perioperative complications such as cardiac arrest, atrial fibrillation, myocardial infarction and heart failure, especially when DEX is used in combination with propofol (Fan et al., 2023; Elgebaly et al., 2020). DEX-assisted anesthesia in craniocerebral surgery can also benefit patients. Fu et al. mentioned that DEX can mitigate oxidative stress, enhance postoperative cognitive function and facilitate postoperative recovery for patients. In addition to the aforementioned effects, DEX also exert protective effects to alleviate pulmonary inflammatory response and oxidative stress, against hepatic I/R injury and maintain the integrity of the intestinal barrier in patients undergoing gastrointestinal surgery (Xie et al., 2020; Fayed et al., 2016; Qi et al., 2022; Figure 1).

## 3 Ferroptosis

Since the term “ferroptosis” was proposed, researches in related fields have grown exponentially in recent years. Ferroptosis is a Regulated Cell Death (RCD) driven by lethal iron-dependent lipid peroxidation, as a result of the imbalance of cell metabolism and redox homeostasis, which is related to various activities such as cell lipid metabolism, iron metabolism and amino acid metabolism. The occurrence of ferroptosis may drive vital organ injuries and degenerative pathologies (Jiang et al., 2021; Liang et al., 2022). Therefore, it is of great significance to conduct in-depth analysis of the mechanism and regulation of ferroptosis, its potential physiological functions, and its roles in diseases and treatments. By summarizing the relevant literature published in recent years, we concluded the main mechanism of ferroptosis as follow.

### 3.1 Ferroptosis and lipid metabolism

Unrestricted lipid peroxidation is a hallmark of ferroptosis. If a specific type of Reactive Oxygen Species (ROS), Polyunsaturated fatty acids (PUFA) phospholipid hydroperoxides, cannot be effectively neutralized and thus accumulate and disrupt plasma membrane integrity. The unsaturation degree of the lipid bilayer is critical in determining cellular susceptibility to ferroptosis. When PUFA phospholipid hydroperoxides is formed and not neutralized rapidly, it can promote the peroxidation of adjacent phospholipids, under the action of Fe^2+^ (Rochette et al., 2022; Li et al., 2020).

Acyl-CoA Synthetase Long Chain Family Member 4 (ACSL4) and Lysophosphatidylcholine Acyltransferase 3 (LPCAT3), as two membrane remodeling enzymes, can drive lipid peroxidation and thereby trigger ferroptosis. PUFA are esterified by activated ACSL4, and then transferred to membrane phospholipids by LPCAT3 to form toxic lipid peroxides (Jiang et al., 2021; Zou et al., 2019). Subsequently, lipid peroxides interact with Fe^2+^, generating peroxide radicals (Pope and Dixon, 2023).

### 3.2 Ferroptosis and iron metabolism

Iron metabolism is mainly regulated by the liver, which maintains systemic iron homeostasis through the production of regulatory factor. The redox cycle between Fe^2+^ and ferric ion (Fe^3+^) enables iron-dependent cofactors to exert their catalytic functions. Fe^3+^ transports into cells via Transferrin Receptor 1 (TfR1), and then reduced to unstable Fe^2+^ by metal reductase (Zeng et al., 2023; Gonciar et al., 2021). Intracellular ferritin participates in regulating iron homeostasis and inhibit iron-mediated oxidative activation and ferroptosis (Sun Y. et al., 2022). Intracellular iron accumulation leads to the release of Fe^2+^ into the cytoplasmic labile iron pool and the occur of Fenton reaction with hydrogen peroxide, generating hydroxyl and hydroperoxide radicals (Chen et al., 2023). Both hydroxyl and peroxide radicals can trigger lipid peroxidation and promote the formation of Advanced Glycation End Products (AGEs). Thus, iron metabolism and lipid metabolism are in crosstalk with each other during ferroptosis. Wang et al. reported that the development of Diabetic kidney disease (DKD) is closely related to iron overload and the incidence of ferroptosis does prompt the development of DKD (Wang et al., 2023).

In addition, iron is important for maintaining mitochondrial function, endoplasmic reticulum stress, and many enzymatic reactions. Lipoxygenases (LOXs), which catalyze the oxidation of polyunsaturated fatty acids, do not contain heme iron, The Fe^2+^ in the catalytic center needs to be oxidized to Fe^3+^ to be activated. Therefore, LOXs activation correlates with the cellular redox state (Rochette et al., 2022; Ru et al., 2024).

### 3.3 Ferroptosis and amino acid metabolism

Nuclear factor-erythroid 2-related factor 2 (NRF2/NFE2L2) is a key protein for maintaining iron homeostasis. Its downstream target genes such as System Xc- (a cystine/glutamate antiporter system, composed of two proteins SLC7A11 and SLC3A2) and Glutathione Peroxidase 4 (GPX4), among others, are involved in correcting redox imbalance in cells (Wang et al., 2020; Friedmann Angeli et al., 2014; Yu et al., 2022). Under steady-state conditions, NRF2 degrades rapidly in the cytosol via the ubiquitin-proteasome pathway. However, under oxidative stress conditions, NRF2 escapes degradation and translocates into the nucleus, thereby increasing ferritin levels and activating Glutathione (GSH) peroxidase (Wang X. et al., 2022; Zhou, 2020; Wu et al., 2023)^.^ Ferritin deficiency induces ferroptosis through downregulation of SLC7A11; whereas increased expression of Ferritin Heavy Chain 1 (FTH1) protects cells from GPX4 inhibitor (RSL3) - induced cellular death (Gao et al., 2015; Fang et al., 2020).

However, Fang et al. (2019) reported that activating the NRF2 pathway will mediate the occurrence of ferroptosis. Researches show that Doxorubicin (DOX) induces NRF2-mediated upregulation of heme oxygenase-1 (HO-1/HMOX1), which causes heme degradation. The accumulation of non-heme iron in serum and cardiac tissue, as free iron accumulate in mitochondria and trigger lipid peroxidation, thereby inducing ferroptosis. They demonstrated that administration of DOX could induce heme degradation through NRF2-mediated upregulation of HO-1, leading to ferroptosis and subsequently cause cardiomyopathy in mice. In contrast to DOX, although multiple studies have shown that DEX can also activate NRF2, this has been associated with the alleviating of iron overload and inhibition of ferroptosis (Lan et al., 2020; Zha et al., 2024; Yan et al., 2024). We suppose this difference may due to the specific levels of intracellular NRF2 and HO-1. However, it is regrettable that there is currently no evidence for quantitative analysis of NRF2 and HO-1 expression levels. Additionally, DEX also activates downstream targets of NRF2, such as System Xc- and GPX4, both involved in correcting the redox imbalance in cells. It is noteworthy that Fang et al. did not mention how DOX affects the expression level of GPX4.

Furthermore, although NRF2-deficient mice are resistant to DOX-induced upregulation of HO-1 and iron accumulation, these mice are highly susceptible to cardiac dysfunction, indicating that the local effects of NRF2 deficiency in the heart may differ from the systemic effects (Li et al., 2014).

GSH is the major antioxidant in mammalian cells, and cysteine is the rate-limiting substrate for GSH biosynthesis. Conditions that impede intracellular cysteine and GSH levels directly affect GPX4 activity (Seibt et al., 2019; Xie et al., 2023). GPX4 is an important inhibitory protein of peroxidation, the core regulator of ferroptosis and has the unique function of reducing PUFA phospholipid hydroperoxides to its counterpart non-toxic phosphatidylinositol (Zeng et al., 2023). Gong et al. (2019) have shown that ferroptosis can be triggered by the inhibition of system Xc- and the inactivation of GPX4. Increased ROS production after GPX4 inhibition sensitizes cells to ferroptosis (Yang et al., 2014). While PUFA phospholipid hydroperoxides level exceeds the reducing capacity of GPX4, phospholipid hydroperoxides accumulates intracellular, which damages the cell membrane.

### 3.4 Endoplasmic reticulum stress (ERS) and mitochondrial dysfunction

Endoplasmic reticulum (ER) plays a crucial role in protein quality control. Conditions such as gene mutations, hypoxia and oxidative stress can induce the occurrence of ERS, leading to the accumulation of unfolded or misfolded proteins within the ER lumen (Zhang J. et al., 2022). An increasing number of studies have found that the activation of ER signaling transduction and ERS can cause ferroptosis (Zhang et al., 2021; Zhang X. et al., 2022).

Protein kinase R-like endoplasmic reticulum kinase (PERK) is a classical pathway of ERS. Wei et al. (2021) reported that ERS can lead to Fe^2+^ accumulation and lipid peroxidation through the PERK/NRF2/HO-1 pathway, thereby inducing ferroptosis. Zheng et al. (2022) mentioned that PERK pathway can also reduce the level of System Xc-through the p53 (a transcription factor) gene, reducing the synthesis of GSH and ultimately promoting ferroptosis. Additionally, Guan et al. (2023) demonstrated that ameliorating ERS through the Cyclic Adenosine Monophosphate (cAMP)/Protein Kinase A (PKA)/Inositol-requiring Enzyme 1 (IRE1) pathway can inhibit ferroptosis, which conversely confirms the relation between ERS and ferroptosis.

Mitochondria, as a highly dynamic organelle, is the primary source of intracellular ROS, which plays a significant role in ferroptosis (Li et al., 2023a). The binding of iron to mitochondrial ferritin prevents ROS production, while the mutation and degradation of mitochondrial ferritin leads to mitochondrial iron overload (Richardson et al., 2010). Lo et al. (Lo and Hannink, 2008) demonstrated that NRF2 can bind to mitochondria, thus indicating and influencing changes in mitochondrial function.

An increasing number of studies have confirmed that there is an interaction between ferroptosis and mitochondrial dynamics, including mitochondrial fission, mitochondrial fusion and mitophagy (Lin et al., 2023; Khatun et al., 2024; Wang LL. et al., 2024). For example, mitophagy exerts a protective effect by clearing dysfunctional mitochondria and reducing the release of ROS (Lin et al., 2023).

### 3.5 The role of ferroptosis in cancer and immunity

Iron is essential for cell proliferation and growth, and tumor cells tend to be more iron-dependent when they are rapidly proliferating. In this case, abnormal iron metabolism causes tumor cells more sensitive to ferroptosis due to higher levels of intracellular iron and ROS (Forciniti et al., 2020; Zhang C. et al., 2022). Studies have shown that iron chelators and drugs that increase iron-mediated toxicity can be used to treat cancer, with the ferroptosis inducers Erastin and RSL3 exerting selective lethal effects on some tumor cells (Chen et al., 2021a). In addition to this, ferroptosis inducers can act synergistically with conventional chemotherapeutic agents (Zhao L. et al., 2022).

NRF2 plays a dual role in tumor progression: lack of NRF2 activity can lead to early tumorigenesis, whereas high basal NRF2 activity can trigger tumor progression and resistance to therapy. The accumulation level of NRF2 in lung cancer cells is higher than that in other cancer cells (Rojo de la Vega et al., 2018; Koppula et al., 2022). It has been shown that the NRF2 signaling pathway is associated with the development of resistance to Sorafenib in hepatocellular carcinoma (Sun et al., 2016). In addition, the relationship between related gene expression and survival outcomes varies in different types of tumors. GPX4 acts as a central inhibitor of ferroptosis in cancer cells, which enhances the cytotoxicity of chemotherapeutic agents in breast cancer as well as the sensitivity to radiotherapy, and thus GPX4 expression level is negatively correlated with the prognosis of breast cancer patients (Song et al., 2021; Ubellacker et al., 2020). However, Dai et al. (2020) have shown that high levels of GPX4 expression have favorable survival outcomes in pancreatic cancer patients.

In a word, ferroptosis plays a crucial role in killing tumor cells and inhibiting tumor growth. Although the mechanism is not fully understood, targeted induction of ferroptosis may become a new cancer treatment strategy.

## 4 Protective effect of DEX in ferroptosis and its mechanism

Previous studies have shown that DEX can attenuate lipid peroxidation and mitochondrial dysfunction to inhibit the occurrence of ferroptosis through multiple pathways such as increasing the activation of NRF2, regulating lipid metabolism and anti-inflammation (Yu et al., 2022; Wang X. et al., 2022; Liu Y. et al., 2021). The specific mechanisms are summarized as follows.

### 4.1 Acyl-CoA synthetase long-chain family member 4

Acyl-CoA synthetase long-chain family member 4 (ACSL4) is an important isoenzyme in polyunsaturated fatty acid metabolism that preferentially utilizes arachidonic acid as a substrate to bind and esterify free long-chain fatty acids to phospholipids, and is a key enzyme in the fatty acid metabolic pathway (Chen et al., 2021b). ACSL4 remodels the phospholipid composition of cell membranes and promotes the interconnection between fatty acid metabolism and ferroptosis, determining cellular sensitivity to ferroptosis. ACSL4 is involved in inducing the ferroptosis process in I/R injury. The inactivation of ACSL4 significantly alleviates tissue damage in mice ferroptosis models (Doll et al., 2017; Ding et al., 2023).

Previous studies have shown that DEX can mitigate I/R-induced damage to multiple vital organs by inhibiting the occurrence of ferroptosis, thereby exerting organ-protective effects (Hou et al., 2023; Sun M. et al., 2022; Hemsinli et al., 2022). Previous studies have found that DEX reduces ACSL4 overexpression in ferroptosis, thereby increasing GPX4 levels and decreasing ferritin (Wang M. et al., 2024; Zhu et al., 2024). Tao et al. (2022) demonstrated that the α_2_-AR antagonist atipamezole, on the other hand, completely reversed these effects, indicating that DEX suppresses ferroptosis by activating α_2_-AR and downregulating ACSL4 signaling.

**FIGURE 2:**
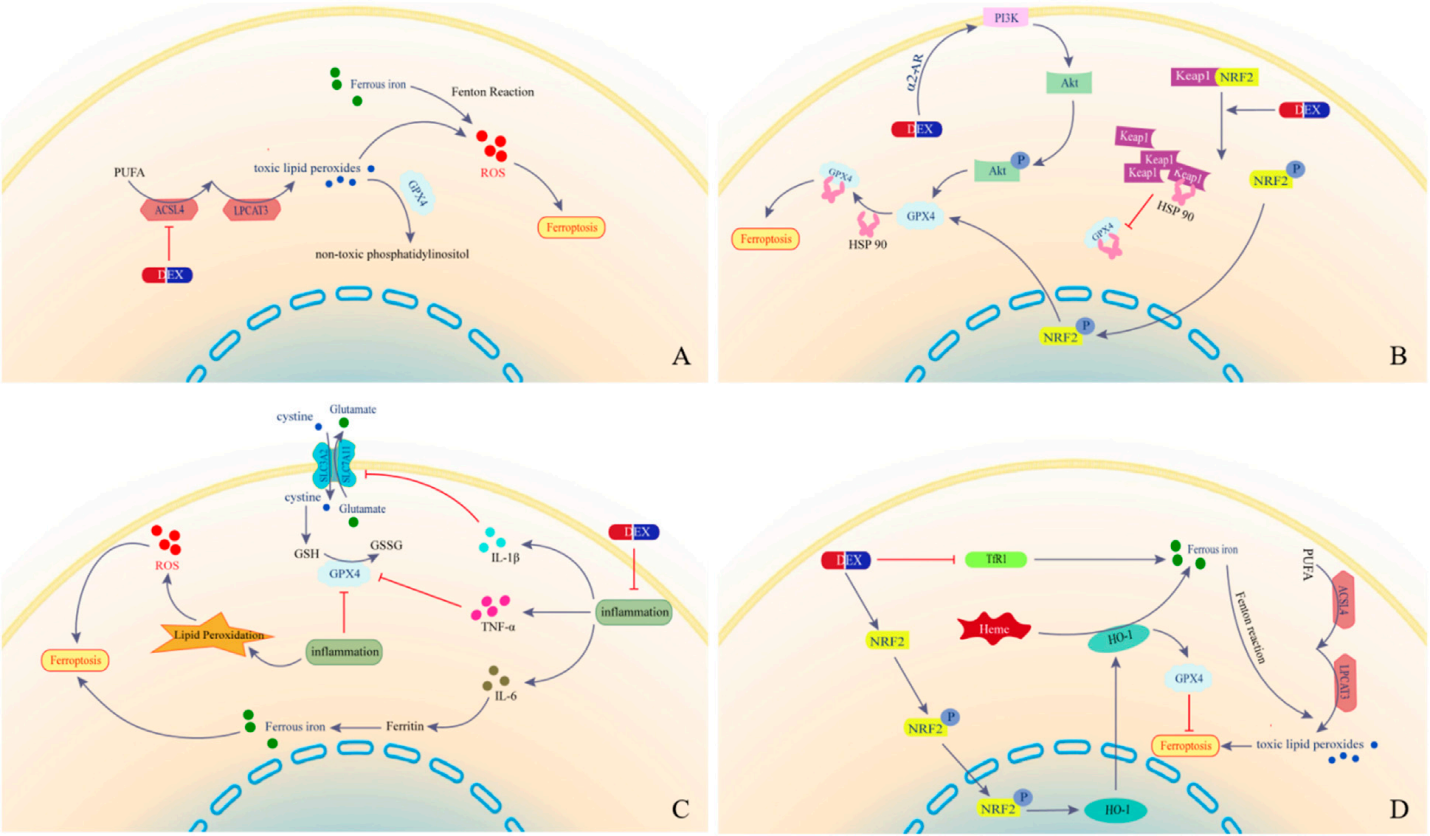
Signal Pathway. **(A)** Acyl-CoA synthetase long-chain family member 4; **(B)** Keap1/NRF2/GPX4 Axis; **(C)** Expression of inflammatory factors; **(D)** HO-1 and Heme. Abbreviations: DEX, Dexmedetomidine; ROS, Reactive Oxygen Species; ACSL4, Acyl-CoA synthetase long-chain family member 4; LPCAT3, Lysophosphatidylcholine Acyltransferase 3; PUFA, Polyunsaturated fatty acids; GPX4, Glutathione Peroxidase 4; ROS, Reactive Oxygen Species; α_2_-AR, α_2_-Adrenergic Receptor; PI3K, Phosphatidylinositol 3-Kinase; Akt, Protein Kinase B; Keap1, Kelch-Like ECH-associated Protein 1; NRF2, Nuclear factor erythroid 2-related factor 2; P, phosphorylated; HSP90, Heat shock protein 90; TNF-α, Tumor Necrosis Factor α; IL-1β, Interleukin-1β; IL-6, Interleukin-6; GSH, Glutathione; GSSG, Glutathione disulfide; HO-1, Heme Oxygenase-1; TfR1, Transferrin Receptor 1.

In addition, ACSL4 regulates inflammation in a manner independent of ferroptosis, and knockdown of ACSL4 leads to a reduction in the production of inflammatory cytokines, which may also be related to the anti-inflammatory properties of DEX (Cui et al., 2021; Zhou X. et al., 2023; Figure 2A).

### 4.2 Kelch-like ECH-associated protein 1 (Keap1)/NRF2/GPX4 axis

NRF2 is a key protein in maintaining iron homeostasis, and its activation can increase ferritin levels and activate glutathione peroxidase, exerting antioxidant functions (Zhou, 2020). The Keap1/NRF2 pathway is a crucial regulatory mechanism of the endogenous redox system. Keap1 is a negative regulator of NRF2, and the modification of cysteine residues in Keap1 can impair its ability to ubiquitinate NRF2 directionally (Wu et al., 2023; Lan et al., 2020). When stimulated by oxidative stress, the Keap1-NRF2 complex is disrupted, leading to a change in the molecular conformation of NRF2 and its translocation from the cytoplasm to the nucleus, where it promotes the transcription of downstream proteins such as GPX4, playing a significant role in antioxidation and anti-ferroptosis (Wang Z. et al., 2022; Li et al., 2023b).

Recent studies also reported a link between the Keap1/NRF2 pathway and mitochondrial function. Activation of Keap1/NRF2 enhances mitochondrial activity and reduces the expression of reduced coenzyme II-associated proteins. NRF2 inhibits mitochondrial oxidative stress by binding to cis-Antioxidant Response Elements (AREs) (Han et al., 2022). Liu Y. et al. (2021) demonstrated that after chronic compression nerve injury, DEX treatment downregulated the expression of Keap1 and increased NRF2 protein levels, thereby activated NRF2 and its downstream signaling pathways. Ding et al. (2024) reported that the myocardial cell showed a significant increase in Keap1 degradation after treated by DEX, which contributed to NRF2 release and nuclear translocation, while Yan et al. (2017) showed that DEX caused conformational changes in the Keap1/NRF2 complex, but no significant changes in Keap1 mRNA levels. Zhou C. et al., 2023) reported that mutual combination of heat shock protein 90 (HSP90) and GPX4 triggers the degradation of GPX4, which in turn induces ferroptosis. As DEX enhances the interaction between Keap1 and HSP90, promotes their binding in the cytoplasm, it can reduce the degradation of GPX4 (Liu Y. et al., 2021).

Although the mechanism by which DEX affects Keap1 via α_2_-AR activation is unclear, studies confirm its role in activating the Keap1-NRF2 pathway and upregulating GPX4 to inhibit ferroptosis.

In addition, a study by Chang et al. (2020) reported that DEX activated the Phosphatidylinositol 3-Kinase/Serine-Threonine Protein Kinase (PI3K/Akt) signaling pathway in an α_2_-AR-dependent manner and enhanced GPX4 expression, while a study by Ma et al. (2023) demonstrated that DEX activated the cAMP/PKA/cAMP-response element binding protein (CREB) pathway, which promoted the expression of GPX4, thereby exerting an inhibitory effect on ferroptosis, suggesting that DEX may act together through a number of different mechanisms in order to increase the level of GPX4 (Figure 2B).

### 4.3 Expression of inflammatory factors

DEX possesses anti-inflammatory properties and improves the prognosis of inflammatory diseases, hence it is widely applied in various inflammation-related conditions such as sepsis and ischemia-reperfusion injury (Zhao S. et al., 2022; Yamaguchi et al., 2023; Li N. et al., 2023). DEX modulates the polarization of M1/M2 phenotype microglia, increasing the polarization of M2-type microglia, which in turn downregulates the expression of anti-inflammatory mediators and exerts its anti-inflammatory effect. Several studies have shown that DEX possesses potent anti-inflammatory properties, significantly decreased the levels of pro-inflammatory cytokines such as Tumor Necrosis Factor α (TNF-α), Interleukin-1 (IL-1) and Interleukin-6 (IL-6), and ROS, increasing the levels of Superoxide Dismutase (SOD) levels, and decreasing oxidative stress levels (Wang et al., 2020; Liu C. et al., 2022; Zhou et al., 2024).

Excessive lipid peroxidation is a significant trigger for ferroptosis, while abnormal inflammatory responses can lead to iron metabolism disorders and an imbalance in redox system. In recent years, an increasing number of studies have shown that the activation of inflammation-related signaling pathways is closely associated with the occurrence of ferroptosis (Chen et al., 2021c; Sun et al., 2020; Chen et al., 2022). Ueda and Takasawa (2018) reported that pro-inflammatory cytokines such as IL-1β, IL-6, and TNF-α can regulate the synthesis of ferritin, thereby affecting iron storage and metabolism. It is worthy of note that IL-6 mediaties the expression of ferritin, which in turn promotes the expression of IL-1β and IL-6 (Wang et al., 2020). The activation of inflammation is accompanied by oxidative stress, which can lead to further dysfunction of the redox system and damage of tissue. Yao et al. (2020) reported that in osteoarthritis (OA), IL-1β can inhibit the expression of ferroptosis markers SLC7A11 and GPX4, and increase the expression of ACSL4. Additionally, TNF treatment of cells leads to sustained downregulation of GPX4, which is essential for the production of lipid mediators (Latchoumycandane et al., 2012).

Nuclear Factor kappa-light-chain-enhancer of activated B cells (NF-κB) is recognized as a central node in inflammation, playing a crucial role in both inflammatory and innate immune responses. TNF-α can activate the NF-κB signaling pathway, interact with HO-1, enhance cellular inflammatory responses, and impact iron metabolism (Guan et al., 2020; Kou et al., 2013). In addition, the activation of Mitogen-Activated Protein Kinase (MAPK) pathway-dependent inflammation is also associated with ferroptosis. Zhu et al. (2021) reported that activation of the MAPK pathway promotes the production of pro-inflammatory cytokines IL-1β, IL-6, and IL-18, reduces the expression of GPX4, and triggers the occurrence of neuro inflammation and ferroptosis (Figure 2C).

### 4.4 HO-1 and iron overload

HO-1 acts as a downstream factor of NRF2 and is upregulated by nuclear translocation of NRF2. HO-1 may play a dual role in ferroptosis. On one hand, the NRF2/HO-1/GPX4 axis has been demonstrated to be a primary defense mechanism against ferroptosis in various diseases. Previous studies have shown that HO-1/biliverdin/carbon monoxide is involved in the progression of antioxidant stress and anti-inflammatory (Wang et al., 2023). On the other hand, HO-1-mediated heme degradation is also a significant source of intracellular iron. Overexpression of HO-1 can lead to excessive iron, which is detrimental to redox balance and can further enhance the production of inflammatory factors (Fang et al., 2019; Qiao et al., 2023). Iron is crucial for physiological processes such as heme synthesis, but excess free iron can catalyze the formation of free radicals through the Fenton reaction, leading to oxidative stress and cellular damage (Jomova and Valko, 2011). Multiple previous studies have demonstrated that DEX treatment can significantly enhance protein levels of HO-1, alleviate oxidative stress, and thereby inhibit ferroptosis, exerting a protective effect (Han et al., 2022; Luo et al., 2024; Li et al., 2022; Hou et al., 2024). On the other hand, Lan et al. (2020) reached the opposite conclusion, indicating that DEX reduced the levels of NRF2 and HO-1 in rats treated with acetic acid. Even so, DEX still provided significant anti-inflammatory and antinociceptive effects.

The Mechanistic Target of Rapamycin (mTOR) axis influences iron metabolism by regulating TfR1, thereby maintaining intracellular iron balance, while high intracellular Fe^2+^ levels lead to the inhibition of mTOR expression (Han et al., 2020; Baba et al., 2018). Qiao et al. (2023) demonstrated that DEX can downregulate TfR1 protein expression and inhibit the increase of Fe^2+^ level by modulating the mTOR-TfR1 signaling pathway. Liu MJ. et al. (2022) also showed that DEX significantly reduces TfR1 protein expression levels and improves mitochondrial structure and function. Since mTOR is one of the most common downstream effectors of Akt, this regulatory effect of DEX on iron homeostasis may also be associated with the activation of the PI3K/Akt pathway (Figure 2D).

### 4.5 Other potential effects of DEX

DEX may improve mitochondrial function by down-regulating lactylation levels. She et al. (2024) reported that DEX suppresses lactate production by down-regulating the lactylation level of Malate Dehydrogenase 2 (MDH2) K241 to improve mitochondrial function and attenuate ferroptosis.

DEX inhibits the activation of the transcription factor Sp1. Qiu et al. (2020) reported that DEX reduces the phosphorylation levels of c-Jun N-terminal kinase (JNK) and Signal Transducer and Activator of Transcription 4 (STAT4), and regulate iron metabolism through the JNK/Sp1 and STAT4/Sp1 pathways, thereby inhibiting the occurrence of ferroptosis.

DEX can also alleviates cardiomyocyte ferroptosis by inhibiting the expression of Histone Deacetylase 2 (HDAC2) and further modulating the HDAC2/Ferroportin 1 (FPN) pathway (Fu et al., 2025).

## 5 Summarize

### 5.1 Mechanisms

Based on the previous studies, we clarified the protective effect and mechanism of DEX in inhibiting ferroptosis, mainly included reducing ACSL4 overexpression, decreasing the expression of inflammatory factors and increasing NRF2 protein levels and the expression of HO-1.

Previous studies have demonstrated that the neuroprotective effects of DEX are mediated through α_2_-AR (Ma et al., 2004; Paris et al., 2006). Many previous studies have shown that α_2_-AR antagonist such as atipamezole can reverse the protective effect induced by DEX in ferroptosis, which indicate that DEX downregulating ACSL4 signaling and alleviating proapoptosis or apoptosis factors increases through the mediation of the α_2_-AR (Tao et al., 2022; Pan et al., 2016).

### 5.2 Controversy

Although many previous studies suggest that DEX activates the PI3K/Akt signaling pathway through an α_2_-AR-dependent manner (Chang et al., 2020; Qiao et al., 2023), Yan et al. (2017) has drawn the opposite conclusion. While investigating the effects of DEX on lipopolysaccharide (LPS)-induced lung injury in Wistar rats, they found that despite DEX improves pulmonary oxygenation and increases NRF2 expression in the lung tissue, it failed to alleviate the inhibitory effect of LPS on the Akt phosphorylation ratio. This may be attributed to variations in the dose and administration route as Chang et al. (2020) treat the rats with 100 μg/kg DEX by intraperitoneal injection while Ma et al. (2004) treat the rats with a total dose of 7.5 μg/kg DEX intravenously. Another possibility is that DEX exerts its protective effect in different organs through a different mechanism. Even so, Yan et al. observed that DEX upregulated expressions of antioxidant genes, which consistent with previous studies.

Another interesting point is that, although the study by Lan et al. (2020), contrary to most studies, suggested that DEX reduced the protein level of HO-1, it still acknowledged the downregulating effect of DEX on inflammatory factors such as IL-1. How is it happen? Li et al. (2023b) showed that DEX failed to activate the Keap1/NRF2 signaling pathway in normal cells, whereas in LPS-stimulated cells, DEX significantly increased the expression levels of NRF2 and HO-1. Since LAN et al. did not measure oxidative stress-related indicators (such as malondialdehyde or SOD). Therefore, it remains unclear whether the stimulation of acetic acid was sufficient to enable DEX to activate the Keap1/NRF2 pathway. It is also worth noting that in the study of Lan et al., DEX was administered via intrathecal injection, while the other studies preferred to use intraperitoneal or intravenous injection.

### 5.3 Limitations

Several studies have linked DEX’s effects to α_2_-AR activation through the use of α_2_-AR antagonist (Tao et al., 2022; Zhu et al., 2021; Fu et al., 2025). However, most studies have not validated this. Whether DEX’s ferroptosis regulation could occur independently of α_2_-AR still requires further investigation. Also, due to significant variations in animal models (mice or rats), routes of administration (intraperitoneal or intravenous), dosages used and experimental period across different studies, our understanding of the dose-dependence of DEX remains incomplete. Experiments with larger sample sizes are still needed. However, it is unequivocal that DEX can exert inhibitory effects within the ferroptosis pathway through a variety of different mechanisms.

Tang Z. et al. (2021) mentioned that the overexpression of HO-1 is associated with the occurrence of ferroptosis. However, it is regrettable that in the references covered by our review, the expression levels of HO-1 were not quantitatively measured, but only qualitatively assessed.

In addition, by carefully reviewing and summarizing recent studies, we found that previous researches on the role of DEX in ferroptosis remain largely confined to animal experiments. Therefore, despite the persisting concern that DEX may not be suitable for extrapolating findings from rodent studies to humans, there is no doubt regarding its safety in clinical applications. Whether DEX can also exert organ-protective effects by inhibiting ferroptosis in the human body needs further research, DEX may provide new insights and directions for the prevention and treatment of ferroptosis-related diseases.
